# Phylogeny and Evolution of Pharmacophagy in Tiger Moths (Lepidoptera: Erebidae: Arctiinae)

**DOI:** 10.1371/journal.pone.0101975

**Published:** 2014-07-18

**Authors:** Jennifer M. Zaspel, Susan J. Weller, Charles T. Wardwell, Reza Zahiri, Niklas Wahlberg

**Affiliations:** 1 Department of Entomology, Purdue University, West Lafayette, Indiana, United States of America; 2 Department of Entomology, University of Minnesota, Saint Paul, Minnesota, United States of America; 3 Biodiversity Institute of Ontario, University of Guelph, Guelph, Ontario, Canada; 4 Laboratory of Genetics, Department of Biology, University of Turku, Turku, Finland; 5 Bell Museum of Natural History, University of Minnesota, Minneapolis, Minnesota, United States of America; Institut National de la Recherche Agronomique (INRA), France

## Abstract

The focus of this study was to reconstruct a phylogenetic hypothesis for the moth subfamily Arctiinae (tiger moths, woolly bears) to investigate the evolution of larval and adult pharmacophagy of pyrrolizidine alkaloids (PAs) and the pathway to PA chemical specialization in Arctiinae. Pharmacophagy, collection of chemicals for non-nutritive purposes, is well documented in many species, including the model species *Utetheisa ornatrix* L. A total of 86 exemplar ingroup species representing tiger moth tribes and subtribes (68 genera) and nine outgroup species were selected. Ingroup species included the most species-rich generic groups to represent the diversity of host-plant associations and pharmacophagous behaviors found throughout Arctiinae. Up to nine genetic markers were sequenced: one mitochondrial (COI barcode region), one nuclear rRNA (D2 region, 28S rRNA), and seven nuclear protein-coding gene fragments: elongation factor 1-α protein, wingless, ribosomal protein subunit S5, carbamoylphosphate synthase domain regions, glyceraldehyde-3-phosphate dehydrogenase, isocitrate dehydrogenase and cytosolic malate dehydrogenase. A total of 6984 bp was obtained for most species. These data were analyzed using model-based phylogenetic methods: maximum likelihood (ML) and Bayesian inference (BI). Ancestral pharmacophagous behaviors and obligate PA associations were reconstructed using the resulting Bayes topology and Reconstructing Ancestral States in Phylogenies (RASP) software. Our results corroborate earlier studies on the evolution of adult pharmacophagous behaviors, suggesting that this behavior arose multiple times and is concentrated in the phaegopterine-euchromiine-ctenuchine clade (PEC). Our results suggest that PA specialization may have arisen early in the phylogeny of the subfamily and that facultative larval pharmacophagous behaviors are the derived condition.

## Introduction

**Figure 1:**
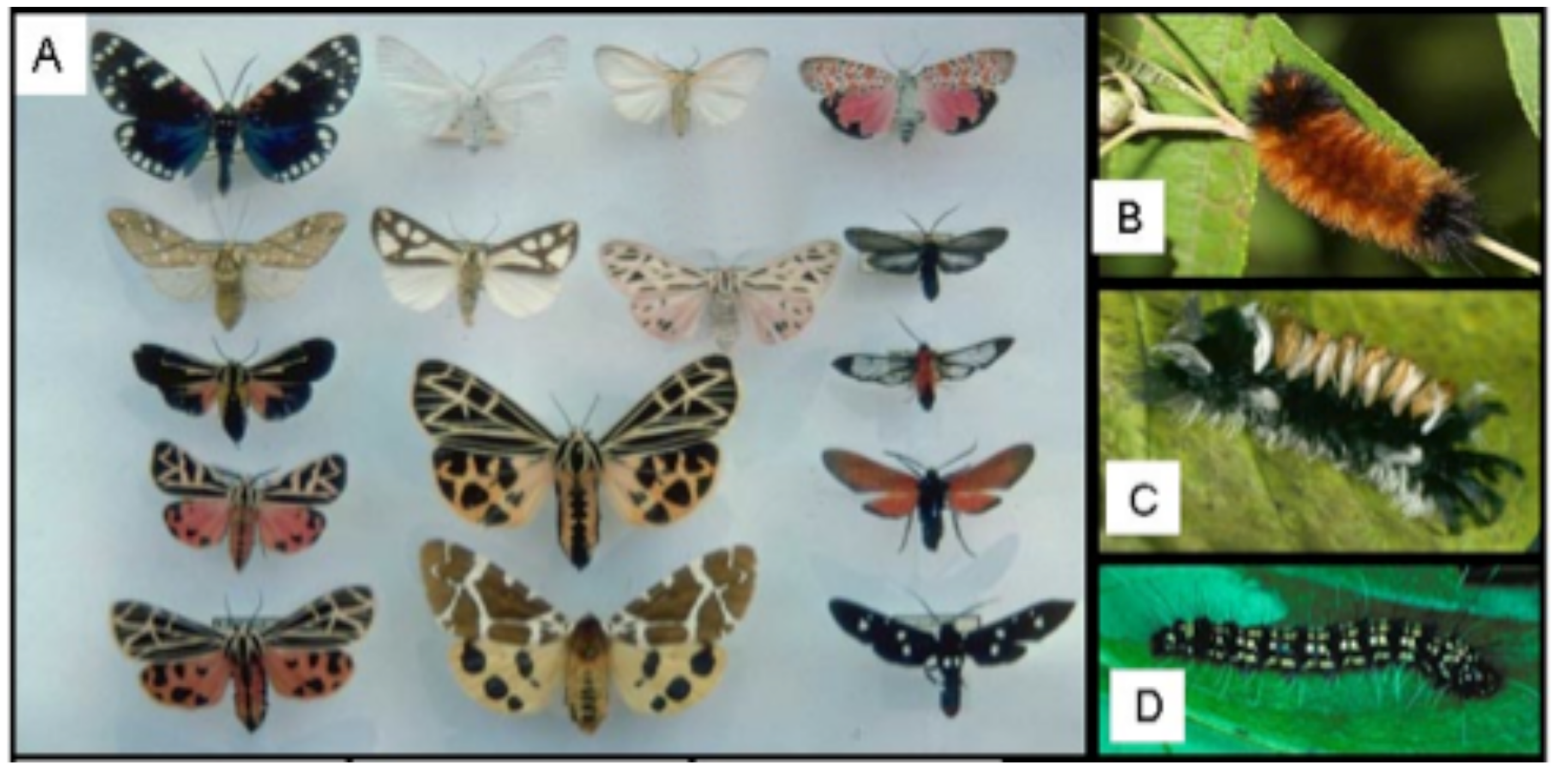
Tiger moth adults/larvae, diversity. **A**. Diversity of adult habitus (photos courtesy: Rebecca Simmons); **B**. Woolly Bear, *Pyrrharctia isabella* (photo courtesy of Bill Conner); **C**. Milkweed tussock, *Euchaetes egle* (photo courtesy of Rebecca Simmons), **D**. Rattle box moth larva, *Utetheisa ornatrix* (photo courtesy Nancy Jacobson).

Arctiinae (tiger moths and woolly bears) are a charismatic moth lineage with a complex evolutionary relationship with plant and fungal chemistries. Comprised of approximately 11,000 species [1], this cosmopolitan group is well known among ecologists and evolutionary biologists for the evolution of bright coloration and spectacular adult mimicry of wasps, beetles and unpalatable moths and butterflies (Figure 1). Both aposematic adults (Figure 1A) and larvae (Figure 1B–D) typically harbor endogenous biogenic amines, like histamines, which are often supplemented with secondary compounds acquired from larval hosts or through *pharmacophagy* - feeding on plants to obtain chemicals rather than nutrients [2]. Some larvae become pharmacophagous when parasitized, and this “self-medication” improves survivorship [3]. Adult pharmacophagy is linked to acquiring chemical constituents of courtship pheromone and nuptial gifts that improve male mating success [2], [4]–[7]. Adult pharmocophagy has been documented in other Lepidoptera, notably Danainae (Nymphalidae) [2]; however, larval pharmacophagy has only been documented in Arctiinae.

Within the mostly phytophagous tribes Arctiini and Amerilini, larvae supplement endogenous defenses by sequestering compounds from their host plants like pyrrolizidine alkaloids (PAs), cardiac glycosides (CGs) or iridoid glycosides (IRs) [4], [8]–[10]. These noxious compounds are of great importance because they have been implicated in severe human and animal diseases [2]. Additionally, through behavioral assays of vertebrate and invertebrate predators, these three classes of compounds (PAs, CGs, and IRs) have been shown to deter predation [11], and of these classes of compounds, the acquisition, sequestration and dissemination of PAs is best understood [12].

Larvae of PA specialist species (e.g., *Utetheisa ornatrix*, *Tyria jacobaeae* (L.) [13], *Creatonotos gangis* (L.)) feed only on PA-containing plants and prefer foliage and seed pods that have the highest concentration of these compounds [12], [14]. Larval PAs are stored in an inactive form for transfer to the adult stage. In some adult males, PAs are transformed into danaidol (and chemical analogs) for dispersal in courtship pheromones [7]. Within the Old World genus *Creatonotos*, PAs are morphogenic; the size and complexity of the male-pheromone dispersing structures (coremata) are dependent upon the PA concentrations consumed by larvae [15]. In the rattlebox moth, *Utetheisa ornatrix,* male mating success depends upon the presence and titer of PAs in his pheromones [16]–[19]. In this species, the males transfer PAs to the female in the spermatophore [20], and the females gain predator protection for themselves and their eggs [21]. In the rattlebox moth, PAs are considered an “honest” good-genes signal of the male's ability to acquire and transport PAs [18], [22]–[23].

In contrast to this PA-obligate system, where PA acquisition and use is linked across the larval and adult stages, several have been chemically documented, and many species have host records of PA-containing plants. In oligophagous or polyphagous species, larvae accept PA-containing plants as part of a broader diet. They do not transfer them to the adult stage or actively collect them as adults. In some cases, the purpose of PA acquisition appears to be medicinal: larvae increase their PA diet when parasitized by endoparasitoids [24]–[25]. Thus, parasitized larvae that self-medicate with PAs tend to have greater resistance to parasitoids and thus higher survivorship than those that do not [26]. The precise mechanism leading to higher survivorship is not understood. Higher titers of PAs have also been shown to delay larval development and incur other fitness tradeoffs [26]–[27]. This behavioral plasticity of facultative pharmacophagy is thought to permit polyphagous larvae to respond to a heterogeneous selective environment [3], [25]. Although larvae facultatively acquire PAs, they do not store them through metamorphosis and transmit these to the pupal and adult stages. Thus, in these cases, the PA pharmacophagy is restricted to the larval stage.

**Figure 2:**
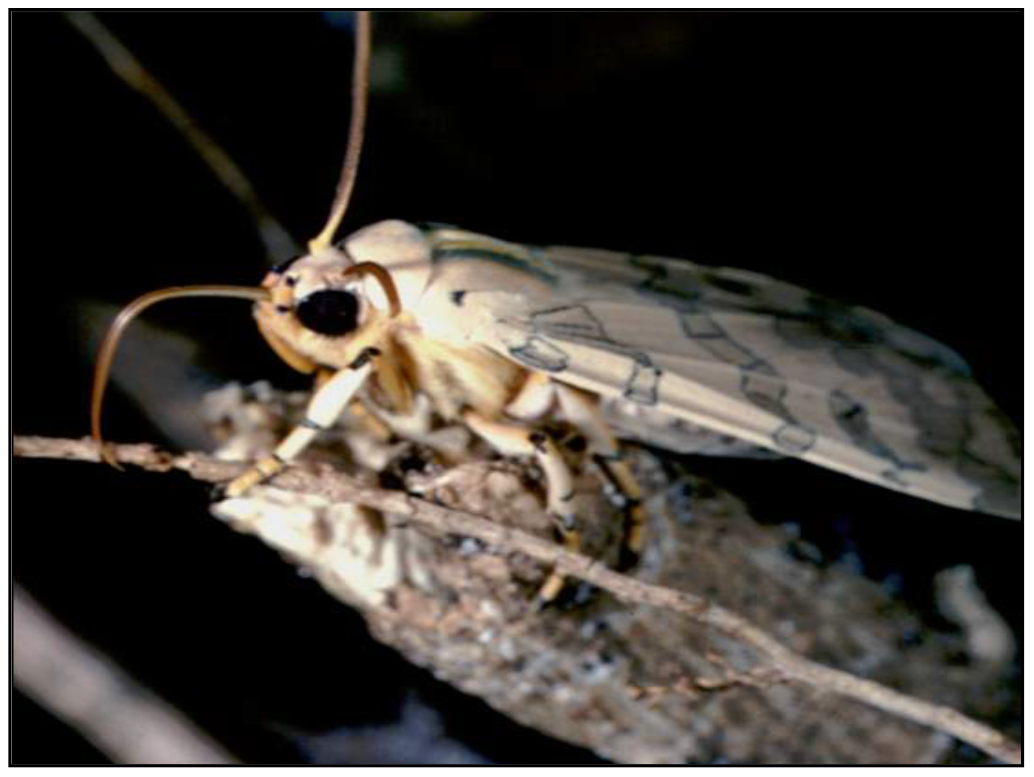
*Halysidota tessellaris* (Smith) imbibing PAs (Photo courtesy Bill Conner).

In contrast, there are several species whose pharmacophagy of PAs only occurs during the adult stage (Figure 2). Many species (typically males) seek out PA-plants to feed at wounds or acquire PAs by regurgitating saliva, dissolving PA crystals on withered plant leaves and re-ingesting the solution [2], [5]–[6], [28]. Males of *Cosmosoma myrodora* (Dyar) have deciduous scales impregnated with PAs that are discharged from a ventral abdominal pouch to cover the female prior to copulation. The female is instantly protected from invertebrate predators by her PA “wedding veil” that is released by the male during courtship [29]–[30]. Many of the species that exhibit adult pharmacophagy occur in the Neotropics, with the exception of the Old World genus *Amerila.*

**Figure 3:**
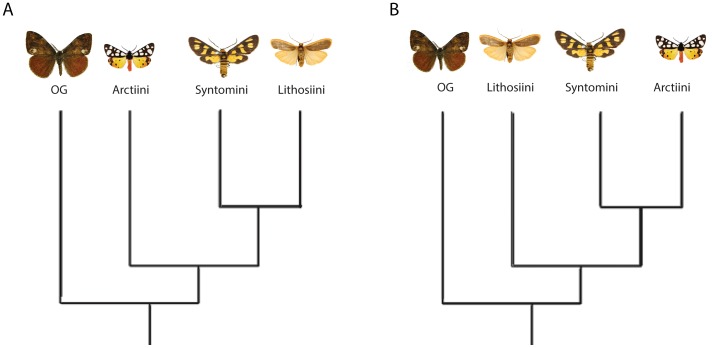
Competing hypotheses for subfamily relationships in tiger moths based on a) Jacobson and Weller 2002 and b) Bendib and Minet 1998.

Despite numerous ecological and behavioral studies, our understanding of arctiine phylogeny and how these pharmacophagous feeding behaviors evolved was limited [10]. A culmination of a series of molecular and morphological studies led to the recent placement of the family Arctiidae as a subfamily Arctiinae of Erebidae [31]–[32] and all taxonomic ranks of older literature is translated to this most recent taxonomy (Table 1) [33]. The only comprehensive phylogenetic reconstruction for Arctiinae was based on adult and immature morphology from 40 exemplar species [34]. The results supported the monophyly of the subfamily and three lineages, tribe Arctiini and the traditional arrangement of a sister group relationship of Lithosiini and Syntomini (Figure 3A). Their tree disagreed with Bendib and Minet [35], who proposed that Syntomini + Arctiini was sister to Lithosiini (Figure 3B). The Old World genus *Amerila* was treated as part of the Arctiini (see review: [9]) until 2009, when a fourth lineage, Amerilini ( =  Rhodogastrinae of Kiriakoff [36]) was resurrected [37]. A recent molecular study [31] suggested that the four lineages should be arranged (Lithosiini(Amerilini(Syntomini + Arctiini), but only 17 arctiinae species were included in the much larger study of Erebidae relationships.

**Table 1:** Examples of recent taxonomic treatments of the former lepidopteran family Arctiidae within the superfamily Noctuoidea.

Bendib & Minet 1998	Kitching & Rawlins 1998; Jacobson & Weller 2002	van Nieukerken et al. 2011 (and references therein)
Noctuoidea	Noctuoidea	Noctuoidea
		Erebidae
	Lymantriidae	Lymantriinae
Arctiidae	Arctiidae	Arctiinae
Amerilinae		
Lithosiinae	Lithosiinae	Lithosiini
Syntominae	Syntominae	Syntomini
Arctiinae	Arctiinae	Arctiini
	Arctiini	Arctiina
	Callimorphini	Callimorphina
	Pericopini	Pericopina
	Phaegopterini	Phaegopterina
	Ctenuchini	Ctenuchina
	Euchromiini	Euchromiina

The focus of this study was to reconstruct a phylogenetic hypothesis of Arctiinae with a large sample of species spanning the diversity of lineages and taxonomy with nine genetic markers. The resulting phylogeny is used to investigate the evolution of pharmacophagy and the pathway to chemical specialization in Arctiinae.

## Materials and Methods

### Taxon Sampling

Our goal was to sample the most species-rich generic groups to represent the diversity of host plant associations and feeding behaviors (e.g., pharmacophagy) found throughout Arctiinae. A total of 86 species from 68 genera representing tiger moth tribes and subtribes were selected based on previous treatments and checklists [31]–[32], [34]–[35], [37]–[39]. We sampled from three traditionally recognized tribes (formerly subfamilies): Lithosiini, Syntomini and Arctiini. We also included Amerilini and the recently proposed Spilosomini [38]. Representatives from former tribes (now subtribes) included Arctiina, Callimorphina, Pericopina, Euchromiina and Ctenuchina [1]. Representative lymantriines, aganaines, and calpines were used as outgroups (nine taxa). Fieldwork related to the present study (e.g., specimen acquisition) did not involve endangered or protected species. Field locations where permits were required with relevant contact information are as follows: USA: Big Cypress National Preserve, FL: collecting and destructive sampling permits were obtained through National Parks Services (Jennifer Stafford and Steve Schulze); COSTA RICA: permits were obtained through the Organization for Tropical Studies (Francisco Campos Rivera); RUSSIAN FEDERATION: Primorsky Krai, permits obtained through the Far Eastern Branch of the Russian Academy of Science (Dr. Vladimir Kononeko); AUSTRALIA: Lamington National Park, Queensland: permits obtained through the Queensland Government Environmental Protection Agency (Jacqui Brock and Ian Bryant). Our exemplars were crossed-checked with their COI sequences (barcode region) in Barcode of Life Data System (BOLD) [40]. A synopsis of the classification, gene sampling and Genbank accession numbers for species included in the study is provided in Table S1.

### DNA Markers, Extraction, PCR and Sequencing

DNA extractions were performed on 1–2 legs (or in some cases abdominal tissue) using the QIAmp Micro DNA Extraction kit (Qiagen, Valencia, CA). The following nine genetic markers were amplified based on previous studies demonstrating their usefulness in resolving evolutionary relationships among lepidopteran insects above and below the family level [41]–[42]: (1) the D2 region of the 28S ribosomal subunit sequence, (2) the barcode region of the COI gene, (3) elongation factor 1-α protein (EF1-α), (4) ribosomal protein subunit S5 (RpS5), (5) wingless (WGS), (6) carbamoylphosphate synthase domain protein (CAD) (7), glyceraldehyde-3-phosphate dehydrogenase (GAPDH) (8), isocitrate dehydrogenase (IDH) and (9) cytosolic malate dehydrogenase (MDH). A total of 6984 bp was obtained for most species. Polymerase chain reaction (PCR) for the majority of the data in this study was performed using ACCUZYME Mix (Bioline) using the following modified recipe: 12.5 µL ACCUZYME Mix (Bioline), 0.5 µL Taq polymerase (Bioline), 1.0 µL of each primer (10 µM), 1.0 µL DNA template, and 9.0 µL ddH2O. PCR amplification of 28S used primer pair 28S-F1 and 28S-R1 [42] and the author's cycling profile, except touchdown PCR was omitted. Amplification of COI was performed with primers Lep-F1 and Lep-R1 [43]. The cycling profile consisted of 3 min at 94°C, 33 cycles of 1 min at 94°C, 1 min at 48°C, and 1 min at 72°C, and a final extension period of 5 min at 72°C. PCR of EF1-α was achieved using the primer pair rcM4 and M46-1 with the modification to M46-1 that the fifth base from the 3′ was made degenerate: G→ (AG) [44]. Thermocycling conditions were 5 min at 95°C, 40 cycles of 94°C for 30 sec, 50°C for 30 sec, and 72°C for 90 sec, with a final extension at 72°C for 10 min. Amplification of RpS5, WGS, CAD, GAPDH, IDH and MDH followed previously described protocols [31]. To facilitate easier high-throughput sequencing, all primer pairs were appended on the 5′ end with the universal T7/T3 primer tails. Successfully amplified products were purified prior to sequencing either the QIAquick PCR Purification kit (QIAGEN) or USB ExoSAP-IT PCR Product Cleanup (Affymetrix) using the manufacturers' protocols. Purified PCR products were sequenced on an ABI 3730xl (Applied Biosystems) using ABI BigDye v. 3.1 Terminator chemistry (PE Applied Biosystems) at the BioMedical Genomics Center (BMGC) at either the University of Minnesota, St. Paul campus or by the company Macrogen Inc (Amsterdam, the Netherlands).

### DNA Sequence Alignment and Phylogenetic Analysis

All protein-coding sequences were aligned using BioEdit v7.2.5 [45] and 28S sequences were aligned using MAFFT v.7.017 [46] implemented in Geneious R6 v.6.1.6 [47]. DNA sequence data was managed and datasets were generated using the web-based software VoSeq [48]. Neighbor-joining and Bayesian analyses of single-gene alignments were performed to test for rogue sequences. If in a single gene analysis, a species placed in a different relationship compared to its placement in the combined, the original sequence data were examined to ensure that contamination had not occurred. In some instances, the sequence amplified but sequenced poorly. If attempts to procure better sequence were unsuccessful, questionable data were omitted.

Maximum likelihood (ML) and Bayesian Inference (BI) analyses were carried out on the combined data set of all markers. The Bayesian Information Criterion (BIC) using Partition Finder v. 1.1.1 [49] was applied to determine the best partitioning scheme for codon positions in protein-coding sequences and corresponding best evolutionary models for the dataset (Table 2). A maximum likelihood (ML) analysis was performed using RAxML-HPC BlackBox v. 7.4.4 [50] on the CIPRES Web Portal at http://www.phylo.org/portal2/ [51]. Bootstrapping runs (1000 replicates) to calculate ML nodal support were also performed on the CIPRES site using the RAxML-HPC BlackBox utility. A partitioned Bayesian analysis of 2.0×10^7^ generations, with 25% of trees discarded as burn-in, was carried out using the MrBayes v.3.2.2 [52]–[53] on XSEDE utility on the CIPRES Web Portal at http://www.phylo.org/portal2/ [51].

**Table 2:** Optimal partitioning scheme selected by PartitionFinder v1.0.1 (Lanfear *et al*. 2012) using the BIC selection criterion.

Partition	Best Partitioning Scheme	Included Nucleotides
1	TVM+I+G	position 1 of CAD, position 1 of MDH
2	GTR+G	position 2 of CAD, position 3 of IDH, position 2 of wingless
3	K80+I+G	position 3 of CAD, position 3 of MDH, position 3 of RpS5, position 3 of wingless
4	GTR+I+G	position 1 of COI, position 3 of Ef1α, position 3 and 1 of GAPDH, position 1 and 2 of IDH, position 1 and 2 of RpS5, position 1 of wingless, 28S
5	K81uf+I+G	position 2 of COI
6	TrN+I+G	position 3 of COI, position 2 of Ef1α, position 2 of GAPDH, position 2 of MDH
7	SYM+I+G	position 1 of Ef1α

The model of evolution for each partition, the number of subsets, parameters, and the log-likelihood for the scheme are included.

### Evolution of PA Acquisition Strategies

An ancestral state reconstruction analysis of PA acquisition strategies was undertaken to elucidate possible feeding shifts and origins of pharmacophagy. Known PA acquisition strategies for tiger moths were divided into the following functional categories: (A) PA-feeding as adult, (B) PA-generalist feeding as larvae, and (C) PA-specialist feeding as larvae. PA acquisition strategies (A–C) were coded for terminal taxa based on documented cases in the primary literature [9]. These categories reflect what is currently known about the biochemical mechanisms of pyrrolizidine alkaloid sequestration (or lack thereof) in tiger moths that are known to be associated with PA plants [12]. Published feeding records suggest PA acquisition strategies in tiger moths are generally conserved at the genus level [9]. Therefore, in cases where published records for a given species were absent, available records for congeners were used (approach as in [54]). In some cases, species exhibited more than one acquisition strategy and thus were assigned more than one state (e.g., *Nyctemera* and *Haploa*). Although other ingroup taxa likely do acquire (and possibly sequester) PA's, detailed documentation of occurrences is limited. For convenience, when assessment of PA use was unavailable, and terminal species for which genus-level data were not available, these species were treated as unknown (Null). Character codings for species in the analysis are included in supplemental materials (Table S2). Tables summarizing PA source records can be found in Conner and Jordan [9] as well as earlier studies [7]–[8].

This data matrix was used to estimate ancestral reconstructions for known PA acquisition strategies following previously published methods [55]. Analyses were based on the resulting trees from the ML analysis of molecular sequence data. We performed a Bayesian binary MCMC (BBM) analysis implemented in RASP (Reconstruct Ancestral State in Phylogenies) [56]–[57] for 20,000,000 generations with sampling at every 100 generations. This method calculates the frequencies of ancestral distributions, ranges or other traits at each node and averages them over all sampled trees [56]–[57]. The fixed JC model and null distribution were used. The maximum number of feeding categories was set at three (with combinations of distributions allowable). The RASP analysis was rooted with the same root for the phylogenetic estimation, a non-PA feeder *C. thalictri* (Erebidae; Table S2).

## Results

### Phylogenetic Analyses

**Figure 4:**
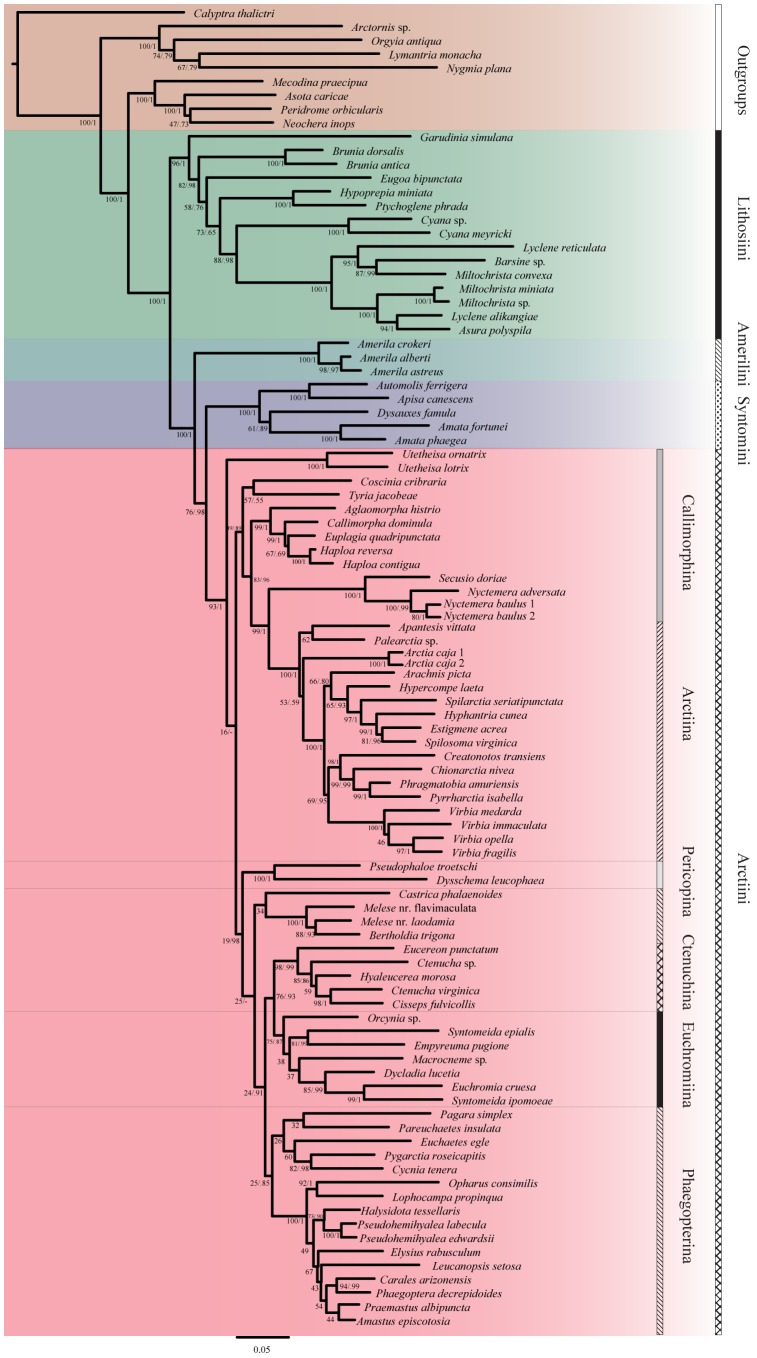
Phylogenetic hypothesis for the subfamily Arctiinae (Noctuoidea, Erebidae) based on maximum likelihood (ML) analysis, along with outgroups. Clades representing tribes are colored. Support values (ML Bootstrap/PP posterior probability) are shown next to the branches.

In the analyses of the combined dataset, BI and ML approaches produced congruent results. The BI and ML analyses recovered a well-supported, monophyletic Arctiinae (bootstrap [BS]  = 100, posterior probability [PP]  = 1) (Figure 4). A clade comprising aganaine genera was sister to it with strong support for this relationship in both analyses (BS  = 100, PP  = 1). Within Arctiinae, four clades corresponding to the tribes Lithosiini, Amerilini, Syntomini and Arctiini were recovered. Tribe Lithosiini placed as sister to remaining arctiines (Figure 4) and its monophyly was strongly supported (BS  = 96, PP  = 1). Within Lithosiini, highly structured, well-supported clades were recovered. Genera *Lyclene* and *Miltochrista* were not recovered as monophyletic (Figure 4), but this result was not unexpected given a much larger study of Lithosiini that included multiple species from these genera [58]. Tribe Amerilini (BS  = 100, PP  = 1) was recovered and placed as sister to the remaining arctiines (BS  = 100, PP  = 1). Support for the clade comprising Arctiini and Syntomini in the BI analysis was high (PP  = .98) and moderate in the resulting ML tree (BS  = 76) (Figure 4). However, support for tribe Syntomini was strong in both analyses (BS  = 100, PP  = 1).

The tribe Arctiini received high support [BS  = 93, PP  = 1], however the genus *Utetheisa*, which has been associated with *Callimorpha* and related genera, differed substantively in its placement in the two analyses. In the ML analysis, *Utetheisa* placed as sister to the rest of Arctiini (Figure 4). In the BI analysis, *Utetheisa* placed within the clade comprising pericopines, phaegopterines, euchromiine and ctenuchine species (PP  = .98) with the novel association of genera *Pagara* and *Castrica* (PP  = .90) (Figure S1). This unexpected result is discussed below.

With the exception of *Utetheisa*, the two analyses agree on the remaining large clades and many of the generic associations. Callimorphina grade into Arctiina in both analyses (Figure 4). However, a large clade is recovered comprising *Callimorpha* and related genera ( =  subtribe Callimorphina). Another clade comprising *Nyctemera* + *Secusio*, was also recovered (BS  = 100, PP  = 1). Subtribe Arctiina was recovered with high support (BS  =  100, PP  = 1). The BI or ML analyses did not recover Spilosomina exclusive of Arctiina [38].

Within Arctiini, a second large clade included the remaining subtribes: Pericopina, Phaegopterina, Ctenuchina and Euchromiina, and this clade was weakly supported in the ML while strongly supported in the BI (BS  = 19, PP  = .98). Within this clade (Figure 4), there was strong support for Pericopina (BS  = 100, PP  = 1), a small generic cluster of *Melese* and *Bertholdia* (BS  = 100, PP  = 1), and a *Eucereon-Ctenucha* clade (BS  = 98, PP  = .99). Euchromiina was recovered with moderate support in the ML analysis (BS  = 75, PP  = .87). Phaegopterina was not recovered as monophyletic in either analysis, but this result was not surprising given prior studies also lacked support for the monophyly of the subtribe [34], [59]. Remaining structure was either not well supported or conflicted between the two analyses (although nodal support was typically weak).

### Ancestral State Reconstructions of PA Acquisition Strategies

**Figure 5:**
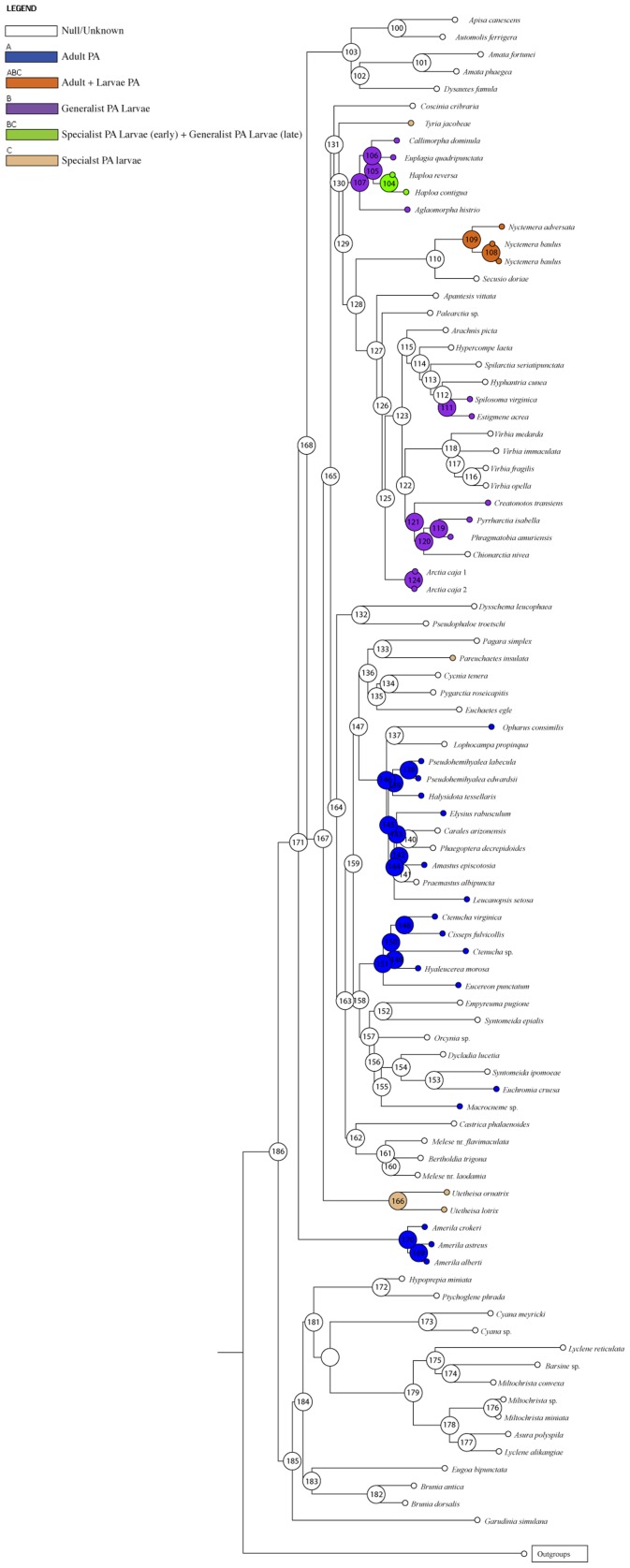
Summary of Bayesian ancestral state reconstruction analysis for major arctiine lineages optimized on the ML topology, implemented in the program RASP (Yan *et al*., 2011). Ancestral PA acquisition strategy reconstructions with highest marginal probabilities are indicated at each node. Blue  =  Adults associated with PAs; Orange  =  Adults and larvae associated with PAs (e.g., *Nyctemera*); Purple  =  Larvae associated with PAs (generalists); Green  =  Larval obligate early polyphagous late (e.g., *Haploa*); Tan  =  Larvae associated with PAs (specialists).

The resulting ML topology with the most likely reconstructions for PA strategy is shown in Figure 5. Our analysis supports the hypothesis of Weller *et al.* [10] that the incorporation of PA's into the larval or adult diets occurred after the ancestor of lichen feeding lithosiines and ancestor of the Arctiini + Amerilini clade diverged (Figure 5). The analysis does not reconstruct a single origin of larval or adult acquisition nor does it find a progression from larval acquisition early in the evolution of tiger moths with adult feeding arising later [10]. Rather, adult PA feeding arises early with a single origin within Amerilini and at least two independent origins in the clade containing Pericopina, Phaegopterina, Euchromiina and Ctenuchina (Figure 5). Species in the genera *Euchromia* and *Macroneme* are also known to acquire PA's during the adult stage but a significant reconstruction of that state was not recovered for Euchromiina, likely due to unknown behaviors in closely related taxa.

Obligate larval acquisition and sequestration (e.g. *Utetheisa* sp. and *Tyria* sp.) precedes the evolution of the generalist feeding strategy within Callimorphina (Figure 5). The callimorphine genus *Haploa* represents a special case of generalist PA feeding within this subtribe because larvae begin as PA plant specialists and then become polyphagous during later instars [9]. *Nyctemera* species (also Callimorphina) are generalist PA feeders during both larval and adult stages [2], [9], [14], [60], [61]. Generalist PA feeding with supplemental pharmacophagy during the immature stages also dominates within Arctiina and may have three origins within the Arctiina; however, the sampling of species (a fraction of the diversity) and knowledge of these behaviors is such that the pattern should not be over-interpreted.

## Discussion

### Phylogeny

The basal position of Lithosiini is expected based on prior studies [31]. Interestingly, the enigmatic *Amerila* (Amerilini) is recovered as sister to Arctiini and Syntomini. This genus has been previously placed in Phaegopterina [35], [61]–[62]. Its placement as sister to the non-lithosiine tiger moths was first suggested by Zahiri *et al.* [31] in the context of their larger study of Erebidae. They had very limited sampling of one *Amerila* species, three syntomines and nine arctiines, but sampled one mitochondrial marker (COI) and seven nuclear genes for their erebid study of 237 species [31]. In their tree, the node that placed Amerilini as sister to Syntomini and Arctiini had 52% ML bootstrap support compared to 95% support in our results; they did not perform a Bayesian analysis. This placement supports the interpretation that the hearing organ morphology, a notodontid-type tympanum, is independently expressed in *Amerila*, Syntomini, *Creatonotos* (Arctiini, Arctiina) and several phaegopterine genera in Arctiini. Finally, the sister relationship of Syntomini and Arctiini supports the hypothesis of Bendib and Minet [35].

Within Arctiini, the relationships of genera formerly placed in Callimorphina (*Callimorpha*, *Euplagia, Haploa, Nyctemera*, *Secusio*, *Tyria*, and *Utetheisa*) need comment. This enigmatic and relatively small group of about 100 “butterfly-like” species placed in eleven genera has a cosmopolitan distribution [1], [62]. The brightly colored, diurnal members are popular with collectors and many forms, subspecies and subgenera have been described [1]. At least two tribes (now subtribes) have been recognized including Nyctemerina, comprising two genera *Nyctemera* and *Secusio* (*Galtara*) [37] and Callimorphina, whose membership has been highly variable, but centered on *Callimorpha* and *Euplagia* [62]. That generic pairs, like *Nyctemera* and *Secusio*, are supported strongly in our analysis and by prior authors [37], [62]–[63] is not surprising, however, the taxonomic usefulness of a subtribe that only includes two of eleven possible genera and about 100 species is debatable. Regardless, the remaining Callimorphina is recovered as a grade of genera subtending Arctiina whose precise generic relationships will require additional species sampling to illuminate. The instability of the placement of *Utetheisa* is likely due to the effect of missing species diversity in our sample from the Austral-Asian and African regions, as well as poor coverage of gene regions (only RpS5, wingless, COI and 28S were sequenced for this genus, see Table S1). We include one New World *Utetheisa* (*U. ornatrix* L.) and Old World tropics *U. lotrix* (Cramer). In an unpublished dissertation [63], a morphological study of adults and maximum parsimony analysis of the fourteen species found that *U. ornatrix* originated early in the evolution of the genus and was not closely related to the Galapagos *Utetheisa* species. Rather, the species *U. lotrix* and then *U. pulchella* L. placed in the clade as sister to the Galapagos species. Additional *Utetheisa* species and the sister genus *Pitasila* should be sampled. Taking taxonomic action to redefine Callimorphina is not recommended until these relationships are tested with more species.

The subtribe Arctiina (formerly Arctiini [34], [59]) is strongly supported by our data. Although our taxon sampling was not designed to specifically test generic limits, our results do reflect some of the arctiine relationships proposed by Ferguson [64]. For example, *Apantesis* and *Palearctia* form a sister grouping (*Neoarctia*-*Grammia* group), although the relationship did not receive strong support in either analysis. The “*Holomelina*” group of Ferguson [64], now *Virbia* (sensu Zaspel and Weller [65]), was strongly supported as monophyletic in both analyses. This clade of four *Virbia* species robustly placed within the assemblage of genera commonly referred to as the *Spilosoma* Group of Ferguson [64] or Spilosomina (formerly Spilosomini; [37]). The taxonomic usefulness of this subtribe is questionable given the tendency of workers in this group of moths is to create higher taxonomic categories to accommodate the diverse color patterns.

A clade of genera placed in Pericopina, Phaegopterina, Euchromiina and Ctenuchina is recovered (Figure 4) with no ML support (n.s., 19) and with high BI support (PP  = .98). The association of these genera have been supported by numerous morphological studies and faunal works (review [34]), and the low ML support is surprising. This clade represents over half the species diversity in the subfamily. We interpret the low ML support as reflecting missing lineage diversity. Within this clade, the generic groupings reflect general consensus based upon prior studies and taxonomic treatments (e.g., [38], [66]–[68]), although none of these nodes are significantly supported by the ML analysis. Our analyses also recover the *Halysidota* Group of Forbes [69] (ML  = 73, PP  = .90) and a possible “jamming” clade (ML  = 100, PP  = 1) comprised of species (*Bertholdia trigona* + *Melese* spp.) known to disrupt echolocation by their bat predators [70]–[71]. The analyses recover the *Euchaetes* Group of Forbes [69] with the inclusion of *Pagara* in the ML analysis (BS  = 26) and its exclusion in the BI analysis (PP  = .91). The placement of *Pagara* as sister to *Castrica* (Figure S1) in the BI analysis is a novel association. Given the low ML support, we do not place confidence in this alternate arrangement.

### Evolution of PA Acquisition Strategies

Our results suggest numerous shifts to PA pharmacophagy as larvae, as adults and once for both life stages within Arctiini. The first occurrence of PA adult pharmacophagy is within the tribe Amerilini (Figure 5). Many species of the Old World genus *Amerila* are known to be pharmacophagous as adults [72], but their larval feeding behaviors are unknown. Syntomini and *Dysschema* are not associated with PA feeding as either larvae or adults.

Within the grade of Callimorphina genera, the evolution of obligatory PA larval specialists occurs in *Utetheisa*, whose larvae specialize on PA plants in the family Fabaceae (e.g. *Crotalaria*) and in *Tyria jacobeae*, a known biological control agent whose larvae specialize on Asteraceae (*Senecio* spp.). The clade comprising *Callimorpha* + (*Euplagia* + *Haploa*) shows a pattern of larval pharmacophagy (Figure 5) with *Haploa* being an exception. This genus contains some species whose larvae are PA specialists during the early instar stages and then shift to a generalist, pharmacophagous strategy during later instar stages [73], or after hibernation [13]. It is unclear whether this pattern of PA acquisition has associated evolutionary costs or advantages. However, it is known that at least one *Haploa* species, *H. clymene* (Brown), uses PA-plant derivatives in courtship behaviors that are similar to other PA-plant feeding larvae [73]. *Nyctemera* comprises species that are obligate PA feeders as larvae [2], [61], [69] and are also facultative PA feeders as adults (Figure 5; [2]).

Generalist larval feeding on PA plants could be the ancestral condition for the Arctiina clade (Figure 5). This habit appears to be widespread across the subtribe as it occurs in all generic groups except *Virbia*; the apparent absence of PA feeding in *Virbia* could however, be attributed to scant feeding records for the group. The fall webworm moth, *Hyphantria cunea*, is known to feed on over 400 species of plants [13], [74], although its associations with PA plants are not well documented. The salt marsh moth, *Estigmene acrea*, a well-known vegetable pest species [75]–[76], is pharmacophagous as larvae [77]. Species of *Spilosoma* are highly polyphagous as larvae with occasional documentation of PA pharmacophagy [78]. Other genera such as *Apantesis*, *Arachnis*, *Pyrrharctia* are all considered general feeders that likely facultatively switch between PA and non-PA host plants [3]. In summary, larval pharmacophagy evolves at least twice (Figure 5) in this part of the tree, obligate larval PA feeding evolves three times (Figure 5) and adult pharmacophagy evolves once in *Nyctemera* (Figure 5).

Only adult pharmacophagy has been documented for species in Pericopina, Euchromiina and Ctenuchina, but verified larval host records are rare. Adult pharmacophagy originates three times with cases present in all three subtribes (Figure 5). Within the Phaegopterina, adult PA feeding appears to be most common in the *Halysidota* group with adult acquisition documented for *Leucanopsis*, *Halysidota* and *Pseudohemihyalea* [2], [5]–[6], [79]–[80]. Within the Euchromina, adult PA feeding is derived and restricted to members of genus *Euchromia* (Figure 5). Adult species in this genus have been observed feeding PA plants in the Solomon Islands (Plate 10 [A–C] in reference [11] and feeding on *Heliotropium* plants in East Africa [28]. Adult pharmacophagy is reconstructed as the ancestral condition for Ctenuchina with occurrences documented for *Ctenucha* [80]–[81], *Antichloris* species have been observed pollinating and visiting the seeds and dead leaves of PA plants [6] while *Hyaleucerea* species have been documented visiting and feeding at PA plant baits [5]. Adult pharmacophagy has been documented in other euchromiines and ctenuchines [82] but their inclusion in our analysis was not possible due the unavailability of DNA quality material.

The results from this study indicate that facultative pharmacophagy on PAs as larvae is a derived condition that likely evolved from PA plant specialization (e.g., *Utetheisa*, *Tyria*). This pattern challenges previous hypotheses that PA specialists arose from a PA generalist ancestor [8], [10]. Rather, our topology favors a specialist to generalist trend, as hypothesized by Krasnoff and Roelofs [83]. Other authors have suggested that facultative PA feeding (or more generally, host switching) is in fact a form of specialization allowing larvae to gain nutrition from one or more plant sources and self-medicate from another [12]. Larval pharmacophagous species are also physiologically adapted to deactivate a broad range of PAs [12], [77]. For example, in *E. acrea* (and others), the gustatory system is equipped with specialized cells that stimulate PA feeding [84]–[85]. Singer and Bernays [3] discuss additional possible benefits to a ‘specialized generalist’ lifestyle in reference to tiger moths.

Our results regarding adult pharmacophagy on PA plants confirm earlier studies [5]–[6], [10] that inferred multiple origins of this behavior within the subfamily. In examined pharmacophagous species, this habit is accompanied by apparently unique sensory structures on the mouthparts of adults [86]. This recent study reported a statistically significant increase in the number of putative PA chemoreceptors on the proboscides of PA pharmacophagous adults when compared with non-pharmacophagous adults or adults of species that obtain PAs only during the larval stages [86]. An intriguing possibility is that micromorphology may be used to predict adult pharmacophagous behavior. We consider this study to be an important initial step towards unraveling the intricate evolutionary interactions between tiger moths and their complex associations with pyrrolizidine alkaloids.

## Supporting Information

Figure S1 **Phylogenetic hypothesis for the subfamily Arctiinae (Noctuoidea, Erebidae) based on Bayesian Inference (BI), along with outgroups.** Clades representing tribes are colored. Support values (posterior probabilities) are shown next to the branches.
(PDF)

Table S1 **Complete list of specimens used in the phylogenetic analysis including the collection locality, sample IDs, and the gene fragments that were successfully amplified.** Genbank accession numbers are listed in the last 5 columns by gene region.
(XLSX)

Table S2 **Distribution data for RASP analysis.** A =  Adult collector of PAs, not larval stage, B =  Larva polyphagous PA feeders, C =  Larva obligate PA specialist, N =  Null.
(CSV)

## References

[1] Weller SJ, Dacosta M, Simmons R, Dittmar K, Whiting M (2009) Evolution and taxonomic confusion in Arctiidae. In: Conner WE, editor. Tiger Moths and Woolly Bears: Behavior, Ecology, and Evolution of the Arctiidae.New York: Oxford University Press. pp. 11–30.

[2] Boppré M (1990) Lepidoptera and pyrrolizidine alkaloids exemplification of complexity in chemical ecology. Journal of Chemical Ecology 16: 165–185. 24264905 10.1007/BF01021277

[3] Singer MS, Mace KC, Bernays EA (2009) Self-medication as adaptive plasticity: Increased ingestion of plant toxins by parasitized caterpillars. PLoS One 4: 1–8. 10.1371/journal.pone.0004796 PMC2652102 19274098

[4] Bowers MD (2009) Chemical defenses in woolly bears: sequestration and efficacy against predators and parasitoids. In: Conner WE, editor. Tiger Moths and Woolly Bears: Behavior, Ecology, and Evolution of the Arctiidae. New York: Oxford University Press. pp. 83–102.

[5] Pliske TE (1975a) Attraction of Lepidoptera to plants containing pyrrolizidine alkaloids. Environmental Entomology 4: 455–473.

[6] Pliske TE (1975b) Pollination of pyrrolizidine alkaloid-containing plants by male Lepidoptera. Environmental Entomology 4: 474–479.

[7] Schulz S (2009) Alkaloid-derived male courtship pheromones. In: Conner WE, editor.Tiger Moths and Woolly Bears: Behavior, Ecology, and Evolution of the Arctiidae. New York: Oxford University Press. pp. 145–154.

[8] Conner WE, Weller SJ (2004) A quest for alkaloids: the curious relationship between tiger moths and plants containing pyrrolizidine alkaloids. In: Cardé RT, Millar JG, editors. Advances in Insect Chemical Ecology. New York: Cambridge University Press. pp. 248–282.

[9] Conner WE, Jordan AT (2009) From armaments to ornaments: the relationship between chemical defense and sex in tiger moths. In: Conner WE, editor. Tiger Moths and Woolly Bears Behaviour, Ecology, and Evolution of the Arctiidae. New York: Oxford University Press. pp. 155–172.

[10] Weller SJ, Jacobson NL, Conner WE (1999) The evolution of chemical defences and mating systems in tiger moths (Lepidoptera: Arctiidae). Biological Journal of the Linnean Society 68: 557–578.

[11] Conner WE (2009) Tiger Moths and Woolly Bears: Behavior, Ecology, and Evolution of the Arctiidae. New York: Oxford University Press. 303 p.

[12] Hartmann T (2009) Pyrrolizidine alkaloids: the successful adoption of a plant chemical defense. In: Conner WE, editor. Tiger Moths and Woolly Bears: Behavior, Ecology, and Evolution of the Arctiidae. New York: Oxford University Press. pp. 55–81.

[13] Wagner DL (2005) Caterpillars of eastern North America: a guide to identification and natural history. Princeton: Princeton University Press. 512 p.

[14] Hartmann T, Witte L (1995) Pyrrolizidine alkaloids: chemical, biological and chemoecological aspects. In: Pelletier SW, editor. Alkaloids: Chemical and Biological Perspectives. Oxford: Pergamon Press. pp. 155–233.

[15] Boppré M, Schneider D (1985) Pyrrolizidine alkaloids quantitatively regulate both scent organ morphogenesis and pheromone biosynthesis in male *Creatonotos* moths (Lepidoptera: Arctiidae) Journal of Comparative Physiology A. 157: 569–577.

[16] Conner WE, Eisner T, Vander Meer RK, Guerrero A, Ghiringelli D, et al (1980) Sex attractant of an arctiid moth (*Utetheisa ornatrix*): A pulsed chemical signal. Behavioral Ecology and Sociobiology 7: 55–63.

[17] LaMunyon CW, Eisner T (1993) Postcopulatory sexual selection in an arctiid moth (*Utetheisa ornatrix*). Proceedings of the National Academy of Sciences USA 90: 4689–4692. 10.1073/pnas.90.10.4689 PMC46578 8506319

[18] LaMunyon CW (1997) Increased fecundity, as a functionof multiple mating in an arctiid moth *Utetheisa ornatrix* . Ecological Entomology 22: 69–73.

[19] Iyengar VK, Reeve HK, Eisner T (2002) Paternal inheritance of a female moth's mating preference. Nature 419: 830–832. 12397356 10.1038/nature01027

[20] Dussourd DE, Harvis CA, Meinwald J, Eisner T (1991) Pheromonal advertisement of a nuptial gift by a male moth (*Utetheisa ornatrix*). Proceedings of the National Academy of Sciences USA 88: 9224–9227. 10.1073/pnas.88.20.9224 PMC52686 1924385

[21] Dussourd DE, Ubik K, Harvis C, Resch J, Meinwald J, et al (1988) Biparental defensive endowment of eggs with acquired plant alkaloid in the moth *Utetheisa ornatrix* . Proceedings of the National Academy of Sciences USA 85: 5992–5996. 10.1073/pnas.85.16.5992 PMC281891 3413071

[22] Iyengar VK, Eisner T (1999a) Heritability of body mass, a sexually selected trait, in an arctiid moth (*Utetheisa ornatrix*). Proceedings of the National Academy of Sciences USA 96: 9169–9171. 10.1073/pnas.96.16.9169 PMC17751 10430914

[23] Iyengar VK, Eisner T (1999b) Female choice increases offspring fitness in an arctiid moth (*Utetheisa ornatrix*). Proceedings of the National Academy of Sciences USA 96: 15013–15016. 10.1073/pnas.96.26.15013 PMC24764 10611329

[24] Bernays EA, Singer MS (2005) Taste alteration and endoparasites. Nature 436: 476. 16049466 10.1038/436476a

[25] Singer MS, Bernays EA (2009) Specialized generalists: behavioral and evolutionary ecology of polyphagous woolly bear caterpillars. In: Conner WE, editor. Tiger Moths and Woolly Bears: Behavior, Ecology, and Evolution of the Arctiidae. New York: Oxford University Press. pp. 103–114.

[26] Singer MS, Carrière Y, Theuring C, Hartmann T (2004) Disentangling food choice quality from resistance against parasitoids: diet choice by a generalist caterpillar. The American Naturalist 164: 423–429. 10.1086/423152 15478095

[27] Singer MS, Bernays EA, Carrière Y (2002) The interplay between nutrient balancing and toxin dilution in foraging by a generalist insect herbivore. Animal Behaviour 64: 629–643.

[28] Boppré M (1981) Adult Lepidoptera ‘feeding’ at withered *Heliotropium* plants (Boraginaceae) in East Africa. Ecological Entomology 6: 449–452.

[29] Boada R (1997) Courtship and defense of the scarlet-bodied wasp moth *Cosmosoma myrodora* Dyar (Lepidoptera: Arctiidae) with notes on related Euchromiines. MS Thesis, Winstom-Salem: Wake Forest University.

[30] Conner WE, Boada R, Schroeder F, Eisner T (2000) Chemical defense: Bestowal of a nuptial alkaloidal garment by a male moth on its mate. Proceedings of the National Academy of Sciences USA 97: 14406–14411. 10.1073/pnas.260503797 PMC18931 11114202

[31] Zahiri R, Holloway JD, Kitching IJ, Lafontaine JD, Mutanen M, et al (2012) Molecular phylogenetics of Erebidae (Lepidoptera, Noctuoidea). Systematic Entomology 37: 102–124.

[32] Zahiri R, Kitching IJ, Lafontaine JD, Mutanen M, Kaila L, et al (2011) A new molecular phylogeny offers hope for a stable family level classification of the Noctuoidea (Lepidoptera). Zoologica Scripta 40: 158–173.

[33] van Nieukerken EJ, Kaila L, Kitching IJ, Kristensen NP, Lees DC, et al. (2011) Order Lepidoptera Linnaeus, 1758. In Zhang, Z.-Q. (Ed.) Animal Biodiversity: An outline of higher-level classification and survey of taxonomic richness. Order Lepidoptera Linnaeus, 1758. Zootaxa. 3148: : 212–221.

[34] Jacobson NL, Weller SJ (2002) A cladistic study of the Arctiidae (Lepidoptera) by using characters of immatures and adults. Thomas Say Publications in Entomology. Lanham: Entomological Society of America. 98 p.

[35] Bendib A, Minet J (1998) Female pheormone glands in Arctiidae (Lepidoptera). Académie des sciences 321: 1007–1014.

[36] Kiriakoff SG (1950) Recherches sur les organes tympaniques des Lépidoptères en rapport avec la classification. IV. Notodontidae. Biologisch Jaarbboek (Dodonaea) 17: 66–111.

[37] Dubalatov VV (2009) Reviewing the African tiger moth genera: 1. A new genus, two subgenera and a species list from the expedition to Malawi by V. Kovtunovich and P. Ustjuzhanin in 2008-2009, with further taxonomic notes on South African Arctiinae (Lepidoptera: Arctiidae: Arctiinae). Atalanta 40: 285–301.

[38] Lafontaine D, Schmidt C (2010) Annotated check list of the Noctuoidea (Insecta, Lepidoptera) of North America north of Mexico. ZooKeys 40: 1–239. 10.3897/zookeys.149.1805 PMC3234417 22207802

[39] Schmidt C, Opler P (2008) Revised checklist of the tiger moths of the Continental United States and Canada. Zootaxa 1677: 1–23.

[40] Ratnasingham S, Hebert PD (2007) BOLD: The barcode of life data system. Molecular Ecology Notes 7: 355–364. 18784790 10.1111/j.1471-8286.2007.01678.x PMC1890991

[41] Wahlberg N, Wheat CW (2008) Genomic outposts serve the phylogenomic pioneers: designing novel nuclear markers for genomic DNA extractions of Lepidoptera. Systematic Biology 57: 231–242. 18398768 10.1080/10635150802033006

[42] Lee S, Brown RL (2008) Phylogenetic relationships of Holarctic Teleiodini (Lepidoptera: Gelechiidae) based on analysis of morphological and molecular data. Systematic Entomology 33: 595–612.

[43] Hebert PD, Penton EH, Burns JM, Janzen DH, Hallwachs W (2004) Ten species in one: DNA barcoding reveals cryptic species in the neotropical skipper butterfly *Astraptes fulgerator* . Proceedings of the National Academy of Sciences USA 101: 14812–14817. 10.1073/pnas.0406166101 PMC522015 15465915

[44] Cho S, Mitchell A, Regier J, Mitter C, Poole R, et al (1995) A highly conserved nuclear gene for low-level phylogenetics: Elongation factor-1α recovers morphology-based tree for heliothine moths. Molecular Biology and Evolution 12: 650–656. 7659020 10.1093/oxfordjournals.molbev.a040244

[45] Hall TA (1999) BioEdit: a user-friendly biological sequence alignment editor and analysis program for Windows 95/98/NT. In: Nucleic Acids Symposium Series: 95–98.

[46] Katoh K, Standley DM (2013) MAFFT multiple sequence alignment software version 7: improvements in performance and usability. Molecular Biology and Evolution 30: 772–780. 23329690 10.1093/molbev/mst010 PMC3603318

[47] Geneious version R6 created by Biomatters. Available: http://www.geneious.com/.

[48] Peña C, Malm T (2012) VoSeq: A Voucher and DNA Sequence Web Application. PLoS ONE 7: e39071. 22720030 10.1371/journal.pone.0039071 PMC3373637

[49] Lanfear R, Calcott B, Ho SY, Guindon S (2012) Partitionfinder: combined selection of partitioning schemes and substitution models for phylogenetic analyses. Molecular Biology and Evolution 29: 1695–1701. 22319168 10.1093/molbev/mss020

[50] Stamatakis A (2006) RAxML-VI-HPC: maximum likelihood-based phylogenetic analyses with thousands of taxa and mixed models. Bioinformatics 22: 2688–2690. 16928733 10.1093/bioinformatics/btl446

[51] Miller MA, Pfeiffer W, Schwartz T (2010) Creating the CIPRES Science Gateway for inference of large phylogenetic trees; November 14; New Orleans, LA. pp. 1–8.

[52] Ronquist F, Huelsenbeck JP (2003) MrBayes 3: Bayesian phylogenetic inference under mixed models. Bioinformatics 19: 1572–1574. 12912839 10.1093/bioinformatics/btg180

[53] Ronquist F, Teslenko M, van der Mark P, Ayres DL, Darling A, et al (2012) MrBayes 3.2: efficient Bayesian phylogenetic inference and model choice across a large model space. Systematic Biology 61: 539–542. 22357727 10.1093/sysbio/sys029 PMC3329765

[54] Hwang WS, Weirauch C (2012) Evolutionary history of assassin bugs (Insecta: Hemiptera: Reduviidae): insights from divergence dating and ancestral state reconstruction. PLoS One 7: e45523. 23029072 10.1371/journal.pone.0045523 PMC3460966

[55] Zaspel JM, Zahiri R, Hoy MA, Janzen D, Weller SJ, et al (2012) A molecular phylogenetic analysis of the vampire moths and their fruit-piercing relatives (Lepidoptera: Erebidae: Calpinae). Molecular Phylogenetics and Evolution 65: 786–791. 22796530 10.1016/j.ympev.2012.06.029

[56] Yu Y, Harris AJ, He X (2010) S-DIVA (Statistical Dispersal-Vicariance Analysis): A tool for inferring biogeographic histories. Molecular Phylogenetics and Evolution 56: 848–850. 20399277 10.1016/j.ympev.2010.04.011

[57] Yu Y, Harris AJ, He X-J (2011) RASP (reconstruct ancestral state in phylogenies). Available: http://mnh.scu.edu.cn/soft/blog/RASP/. 10.1016/j.ympev.2015.03.008 25819445

[58] Scott CH, Zaspel JM, Chialvo P, Weller SJ (2014) A preliminary molecular phylogenetic assessment of the lichen moths. Systematic Entomology 39: 286–303.

[59] Kitching IJ, Rawlins JE (1998) The Noctuoidea. In: N.P K, editor. Lepidoptera Handbuch der Zoologie.Berlin: de Gruyter. pp. 355–402.

[60] Benn M, DeGrave J, Gnanasunderam C, Hutchins R (1979) Host-plant pyrrolizidine alkaloids in *Nyctemera annulata* Boisduval: Their persistence through the life cycle and transfer to a parasite. Experientia 35: 731–732.

[61] Holloway JD (1988) The Moths of Borneo: Family Arctiidae, Subfamilies Syntominae, Euchromiinae Arctiinae; Noctuidae misplaced in Arctiidae (*Camptoloma*, Aganainae). Kuala Lumpur: Malaysian Nature Society. 101 p.

[62] DaCosta M, Weller SJ (2005) Phylogeny and classification of Callimorphini (Lepidoptera: Arctiidae: Arctiinae). Zootaxa 1025: 1–94.

[63] DaCosta M (2007) Phylogenetic studies of *Utetheisa* Hubner, the rattle box moth, and other arctiines (Lepidoptera: Noctuoidea: Arctiidae). Ph.D. Thesis, St. Paul: University of Minnesota. 363 p.

[64] Ferguson DC (1985) Contributions toward reclassification of the world genera of the tribe Arctiini, part 1: introduction and a revision of the *Neoarctia*-*Grammia* group (Lepidoptera: Arctiidae: Arctiinae). Entomography 3: 181–275.

[65] Zaspel JM, Weller SJ (2006) Review of generic limits of the tiger moth genera *Virbia* Walker and *Holomelina* Herrich-Schäffer (Lepidoptera: Arctiidae: Arctiinae) and their biogeography. Zootaxa 1159: 1–68.

[66] Watson A, Goodger DT (1986) Catalogue of the Neotropical tiger moths. Occasional Papers on Systematic Entomology 1: 1–57.

[67] Simmons RB, Weller SJ (2002) What kind of signals do mimetic tiger moths send? A phylogenetic test of wasp mimicry systems (Lepidoptera: Arctiidae: Euchromiini). Proceedings of the Royal Society of London, Biological Sciences 269: 983–990. 10.1098/rspb.2002.1970 PMC1690985 12028753

[68] Simmons RB, Weller SJ, Johnson SJ (2012) The evolution of androconia in mimetic tiger moths (Noctuoidea: Erebidae: Arctiinae: Ctenuchina and Euchromiina). Annals of the Entomological Society of America 105: 804–816.

[69] Forbes WTM (1960) The Lepidoptera of New York and neighboring states, part 4: Agaristidae through Nymphalidae including butterflies. Ithaca: Cornell University. 188 p.

[70] Conner WE, Hristov NI, Barber J (2009) Sound strategies: acoustic aposematism, startle, and sonar jamming. In: Conner WE, editor. Tiger moths and woolly bears Behaviour, ecology, and evolution of the Arctiidae. New York: Oxford University Press. pp. 177–192.

[71] Corcoran AJ, Barber JR, Conner WE (2009) Tiger Moth Jams Bat Sonar. Science 325: 325–327. 19608920 10.1126/science.1174096

[72] Häuser CL, Boppré M (1997) A revision of the Afrotropical taxa of the genus *Amerila* Walker (Lepidoptera: Arctiidae). Systematic Entomology 22: 1–44.

[73] Davidson RB, Baker C, McElveen M, Conner WE (1997) Hydroxydanaidal and the courtship of *Haploa* (Arctiidae). Journal of the Lepidopterists' Society 51: 288–294.

[74] Warren LO, Tadic M (1970) The fall webworm, *Hyphantria cunea* (Drury). Arkansas Agricultural Experiment Station Bulletin 759.

[75] Capinera JL (1978) Consumption of sugarbeet foliage by the saltmarsh caterpillar. Journal of Economic Entomology 71: 661–663.

[76] Capinera JL (2001) Handbook of Vegetable Pests. New York: Academic Press. 729 p.

[77] Hartmann T, Theuring C, Beuerle T, Klewer N, Schulz S, et al (2005) Specific recognition, detoxification and metabolism of pyrrolizidine alkaloids by the polyphagous arctiid *Estigmene acrea* . Insect Biochemistry and Molecular Biology 35: 391–411. 15804574 10.1016/j.ibmb.2004.12.010

[78] Rothschild M, Alpin RT, Cockrum PA, Edgar JA, Fairweather P, et al (1979) Pyrrolizidine alkaloids in arctiid moths (Lep.) with a discussion on host plant relationships and the role of these secondary plant substances in the Arctiidae. Biological Journal of the Linnean Society 12: 305–326.

[79] Goss GJ (1979) The interaction between moths and plants containing pyrrolizidine alkaloids. Environmental Entomology 8: 487–493.

[80] Krasnoff SB, Dussourd DE (1989) Dihydropyrrolizine attractants for arctiid moths that visit plants containing pyrrolizidine alkaloids. Journal of Chemical Ecology 15: 47–60. 24271426 10.1007/BF02027773

[81] Beebe W, Kenedy R (1957) Habits, palatability, and mimicry in thirteen ctenuchid moth species from Trinidad. BWI Zoologica 42: 147–158.

[82] Brehm G, Hartman T, Willmott K (2007) Pyrrolizidine alkaloids and pharmacophagous Lepidoptera visitors of *Prestonia amabilis* (Apocynaceae) in a montane rainforest in Ecuador. Annals of the Missouri Botanical Garden 94: 463–473.

[83] Krasnoff SB, Roelofs WL (1990) Evolutionary trends in the male pheromone systems of arctiid moths: evidence from studies of courtship in *Phragmatobia fuliginosa* and *Pyrrharctia isabella* (Lepidoptera: Arctiidae). Zoological Journal of the Linnean Society 99: 319–338.

[84] Bernays EA, Chapman RF, Hartmann T (2002) A highly sensitive taste receptor cell for pyrrolizidine alkaloids in the lateral galeal sensillum of a polyphagous caterpillar, *Estigmene acraea* . Journal of Comparative Physiology A 188: 715–723. 10.1007/s00359-002-0345-3 12397442

[85] Bernays EA, Chapman RF (2001) Taste cell responses in the polyphagous arctiid, *Grammia geneura*: towards a general pattern for caterpillars. Journal of Insect Physiology 47: 1029–1043. 11472766 10.1016/s0022-1910(01)00079-8

[86] Zaspel JM, Coy S, Habanek K, Weller SJ (2013) Presence and distribution of sensory structures on the mouthparts of self-medicating moths. Zoologischer Anzeiger - A Journal of Comparative Zoology 253: 6–10.

