# Supplementary figures and images for: Phylogeny and Evolution of Pharmacophagy in Tiger Moths (Lepidoptera: Erebidae: Arctiinae)
Source: PLoS One. 2014 Jul 18;9(7):e101975. doi: 10.1371/journal.pone.0101975 (PMC4103773; doi:10.1371/journal.pone.0101975)

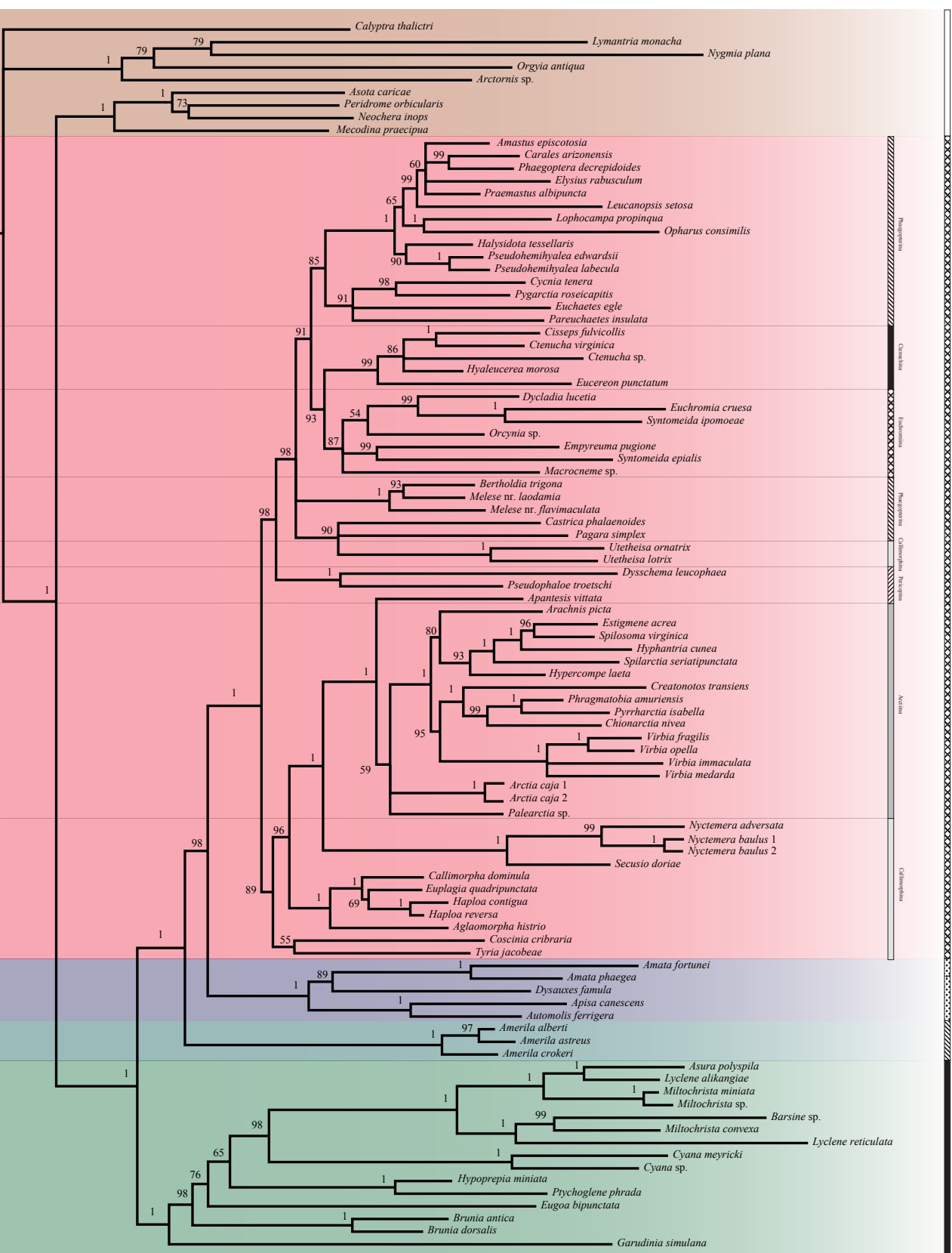

Supplement: Figure S1 — Phylogenetic hypothesis for the subfamily Arctiinae (Noctuoidea, Erebidae) based on Bayesian Inference (BI), along with outgroups. Clades representing tribes are colored. Support values (posterior probabilities) are shown next to the branches. (PDF) [file pone.0101975.s001.pdf]
